# Study on Doppler Spectra of Electromagnetic Scattering of Time-Varying Kelvin Wake on Sea Surface

**DOI:** 10.3390/s22197564

**Published:** 2022-10-06

**Authors:** Tian-Ci Yang, Ye Zhao, Guo-Shan Wu, Xin-Cheng Ren

**Affiliations:** School of Physics and Electronic Information, Yan’an University, Yan’an 716000, China

**Keywords:** Kelvin wake, time-varying, Doppler spectra, sea surface

## Abstract

In general, it is more practical to detect ship wake under the background of a complicated sea state than the ship directly. Thus, in this paper, the Doppler spectra of time-varying Kelvin wake on a time-varying sea surface are numerically investigated by considering the change of ship wake with time in ocean environments. For this purpose, the linear superposition model of a time-varying sea surface and a time-varying Kelvin wake is established. Combined with the facet scattering field model of sea surface and Kirchhoff approximation (KA), the Doppler of the radar scattering echo signal of the time-varying wake on the sea surface is simulated and analyzed under different polarizations, incident angles, ship speeds, and wind speeds, as well as wind directions. It can be observed that the Doppler spectrum changes as the conditions change. This study provides a reference for extracting motion features of ship wakes in a sea clutter environment.

## 1. Introduction

With the development of stealth technology, it has become difficult to directly detect moving ships at sea. Fortunately, the wake induced by a ship crossing the sea surface can last a long time and can extend for several kilometers [1,2,3,4]. The study of scattering characteristics of ship wake is of great significance for ship detection and identification in complex marine environments.

Lord Kelvin was the first to investigate the ship-generated wake and derive the equation of the Kelvin wake model. Because the study of a ship-generated wake is more effective than the direct study of ships, many scholars have conducted various studies on ship-generated wake. In terms of the research on electromagnetic scattering from the Kelvin wakes, Meng et al. [5] calculated the electromagnetic scattering characteristics from the ship-induced Kelvin wake on the rough sea surface by Integral Equation Model (IEM). Rui et al. [6] used the second-order small-slope approximation method to investigate the composite electromagnetic scattering characteristics of the Kelvin wake and the sea surface. Lu et al. [7] used the small-slope approximation method combined with the MPI parallel acceleration algorithm to investigate the electromagnetic scattering characteristics of Kelvin wake. Daniela et al. [8] investigated the SAR imaging of ship wakes in X-band and C-band. Graziano et al. [9] developed an algorithm for estimating ship heading and speed using SAR images, which is based on Radon transform to detect typical wake structures. Zhi-Hua et al. [10] described the process of assessing the electromagnetic field induced by the wake of an undersea-moving slender body. Yu-Xin et al. [11] proposed a hybrid method for separating and reconstructing ship wake systems in the wave elevation and SAR image domains. Yu-Huan et al. [12] proposed a hybrid method to detect linear features of ship wakes in SAR images, and estimated ship heading and speed based on the detection results. Nan et al. [13] realized the location of Kelvin wake by partitioning two-dimensional dynamic sea surface and performing feature selection verification. Yuxin et al. [14] proposed a method combining computational fluid dynamics and electromagnetic imaging to study the effect of underwater motion of an object on electromagnetic scattering from the sea surface. Yinyu et al. [15] proposed a method to study the wake detection of Kelvyn ships in SAR by periodic structure scattering calculation. Fuduo et al. [16] proposed a submarine velocity estimation method based on wake wavelength and a diving depth inversion method based on Kelvin wake Fourier power spectrum. Recently, Yanming et al. [17] proposed a data-driven approach based on dynamic pattern decomposition for detecting, reconstructing, and locating Kelvin wakes on a two-dimensional dynamic sea surface.

Most of the investigation is based on the static model of Kelvin wakes without the time-varying factor that belongs to the traditional model by Lord Kelvin from a long time ago. In previous experiments on the Kelvin wake, both the SAR imaging research of the ship wake and the feature extraction and detection research of the Kelvin wake in the marine environment have focused on the static feature observation of the Kelvin wake, and it is difficult to observe its time-varying characteristics. However, in the complex marine environment, the study of the time-varying characteristics of the Kelvin wake is as important as the research of its static characteristics. In order to research the time-varying properties of Kelvin wakes, Jie et al. [18] proposed a time-varying Kelvin wake model based on the original Kelvin wake model, and its accuracy is verified by a velocity observation experiment. In this paper, based on the time-varying Kelvin wake model, the radar scattering echo of the Kelvin wake induced by ship motion on the time-varying sea surface is studied numerically. 

The rest of this paper is structured as follows. Section 2 outlines the modeling of a time-varying sea surface and a time-varying Kelvin wake. Section 3 introduces the method of calculating the electromagnetic scattering of the sea surface. Section 4 presents simulation results and discussion. Finally, Section 5 forms the conclusions.

## 2. Time-Varying Linear Superposition Model of Sea Surface and Kelvin Wake

### 2.1. Time-Varying Sea Surface Model

In this paper, the Elfouhaily omnidirectional sea spectrum and the Longuet–Higgins directional distribution function are combined to simulate the two-dimensional sea spectrum:

(1) $$W\left(k,\varphi \right)=W_{\mathrm{Elf}}\left(k\right)G_{\mathrm{LH}}\left(k,\varphi -\varphi_{w}\right)/k$$

where

(2) $$W_{\mathrm{Elf}}(k)=k^{-3}(B_{l}+B_{h})$$

(3) $$G_{\mathrm{LH}}\left(k,\varphi \right)=G_{0}(s){\left|\cos\left(\frac{\varphi -\varphi_{w}}{2}\right)\right|}^{2s}$$

(4) $$s=1-\frac{1}{\ln2}\ln\left[\frac{1-\Delta \left(k\right)}{1+\Delta \left(k\right)}\right]$$

(5) $$G_{0}(s)=1/\int_{-\pi }^{\pi }\cos^{2s}\left(\frac{\varphi }{2}\right)d\varphi$$

where *W*_Elf_(*k*) is the Elfouhaily omnidirectional sea spectrum [19], G_LH_(*k*,*φ*) is the Longuet–Higgins directional distribution function, *B_l_* represents gravity wave with longer wavelength, and *B_h_* represents tension wave spectrum with a shorter wavelength. k is the wavenumber, *φ* is the wave number direction angle of the sea waves, and *φ*_w_ is the wind direction. Assuming ***r*** = (*x*, *y*) and ***k*** = (*k_x_*, *k_y_*), according to the linear filtering theory [20], the fluctuation height of a point ***r*** on the sea surface at time *t* can be defined as follows:

(6) $$Z_{s}\left(x,y,t\right)=\frac{1}{L_{x}L_{y}}\sum \sum F\left(k,t\right)\exp(ik\cdot r)$$

**Figure 1:**
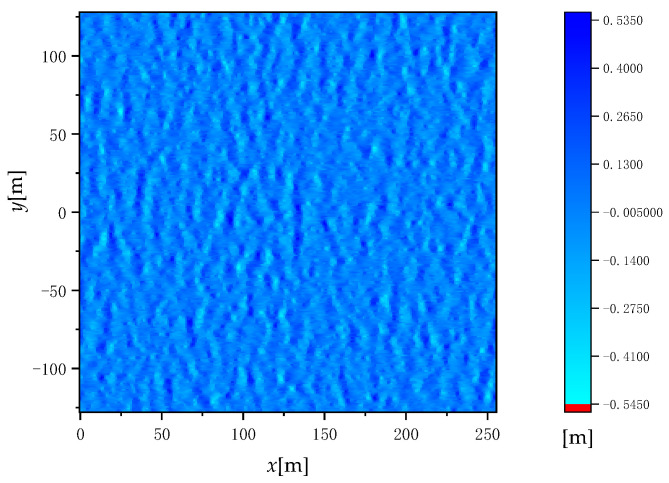
Height of sea surface wave simulated by sea surface modeling method.

In the equation above:

(7) $$\begin{array}{l} F\left(k,t\right)=\\ \gamma \left(k\right)\pi \sqrt{2L_{x}L_{y}W\left(k,\varphi \right)}\;e^{i\omega \left(k\right)t}+\gamma^{*}\left(-k\right)\pi \sqrt{2L_{x}L_{y}W\left(-k,\pi -\varphi \right)}\;e^{-i\omega \left(-k\right)t} \end{array}$$

where *γ*(***k***) is a two-dimensional complex Gaussian random sequence with mean 0 and variance 1 (conforming to the standard normal distribution). $\gamma^{*}\left(k\right)$ is the reverse conjugate of γ(k), and *L_x_* and *L_y_* are the length of the 2D random rough sea surface in the *x*-axis and *y*-axis directions. The height of the sea surface wave simulated by this sea surface modeling method is shown in Figure 1. The simulation parameters of Figure 1 are wind speed u = 5 m/s, time *t* = 0, and wind direction *φ*_w_ = 0°.

### 2.2. Time-Varying Kelvin Wake Model

**Figure 2:**
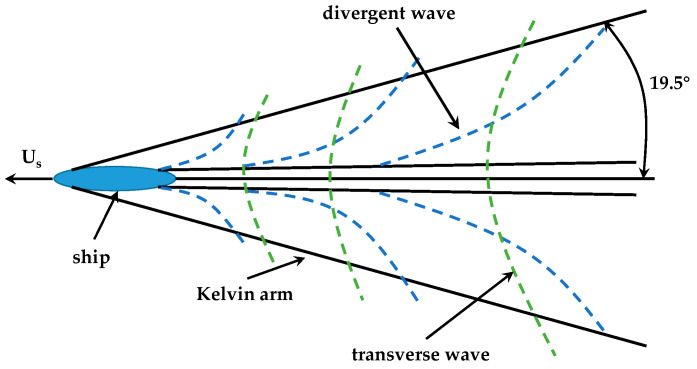
Schematic diagram of Kelvin wake.

The Kelvin wake is composed of divergent waves and transverse waves. The schematic diagram of Kelvin Wake is shown in Figure 2. The divergent waves spread to both sides of the ship, while the transverse waves mainly propagate to the rear of the ship. Where they meet, the two waves coherently form a sharp head wave. Because the wavelength of the sharp head waves is too short, the wavefront of each sharp head wave cannot be distinguished separately, and all the wavefronts are visually connected as a straight line and appear as a bright line, which is called the Kelvin arm. The angle of the Kelvin arm is about ±19.5°.

Based on Lord Kelvin’s theory, the wave elevation propagating by a ship moving at a speed U_s_ in the *x* direction can be obtained as below:

(8) Zk(x,y)=Re∫−π/2π/2A(θ)exp[−ikksec2θ(xcosθ+ysinθ)]dθ

where *k_k_* = g/U_s_^2^, g is the acceleration due to gravity, and *A*(*θ*) is the free spectrum that depicts the ship’s characteristics, which can be written as follows:

(9) $$A(\theta )=\frac{2k_{k}}{\pi }\sec^{3}\theta \int \int \frac{\partial y(x,z)}{\partial x}\exp[k_{k}\sec^{2}\theta (ix\cos\theta +z)]dxdz$$

where *y*(*x*,*z*) is the hull equation of the ship. If we consider a simple hull shape with parabolic waterlines, and if it is a wall-sided ship with draft depth *d*, then [7]:

(10) $$y(x,z)=\{\begin{array}{cc} b(1-\frac{x^{2}}{l^{2}}), & -d\leq z\leq 0,-l<x<l\\ 0, & z<-d \end{array}$$

where *b* is the half-beam, and *l* is the half-length of the ship.

**Figure 3:**
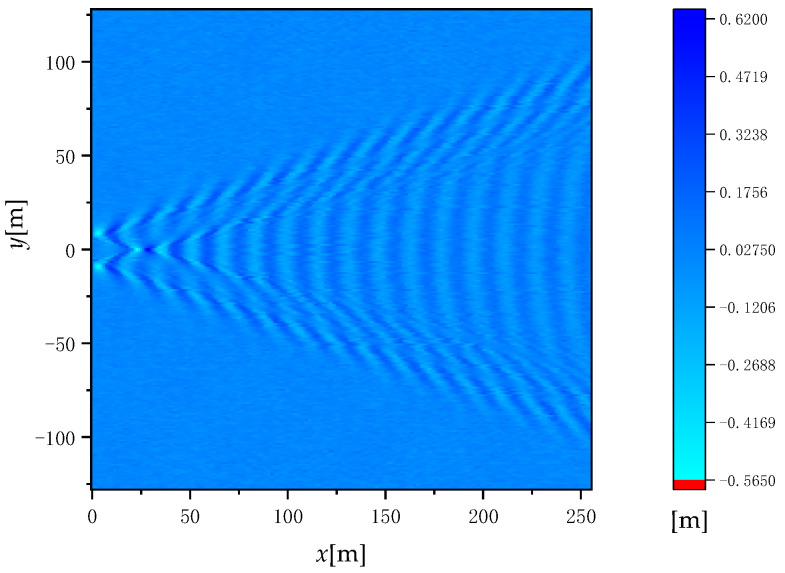
Wave height of the time-varying Kelvin wake.

Considering the effect of time variation on Kelvin wake, based on the original Kelvin wake formula, geometric time-varying characteristics, and fluid dynamics equation, the time-varying Kelvin wake model can be defined as follows [18]:

(11) Zk(x,y,t)=Re∫−π/2π/2J0(kktan(19.5o)Ust)A(θ)exp[−ikksec2θ((x+Ust)cosθ+ysinθ)]dθ

where *J*_0_ is a zero-order Bessel function. The premise of the time-varying model is that the ship is in a uniform rectilinear motion. Table 1 gives the parameters of the ship to simulate the Kelvin wake. Figure 3 shows the wave height of the time-varying Kelvin wake for time *t* = 0 and ship speed *U*_s_ = 5 m/s.

**Figure 4:**
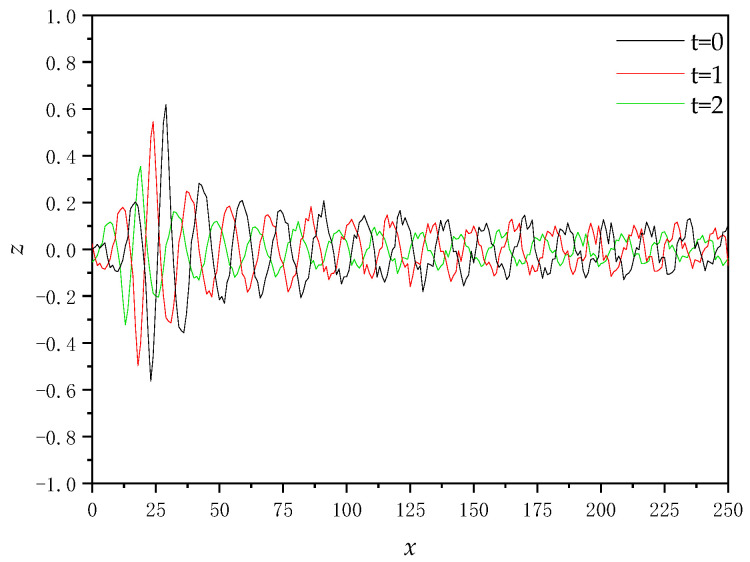
Cross-sectional plot of ship’s induced wake wave height.

Figure 4 shows the cross-sectional plot of the ship’s induced wake wave height for ship speed *U*_s_ = 5 m/s.

It can be seen from Figure 4 that the height of the wake wave induced by the ship gradually decreases with the passage of time, and the movement direction is consistent with the ship.

### 2.3. Linear Superposition of Kelvin Wake and Sea Surface

The Kelvin wake can be linearly superimposed with the sea surface, and the superimposed wave height takes the following form:

(12) $$Z(x,y,t)=Z_{s}(x,y,t)+Z_{k}(x,y,t)$$

**Figure 5:**
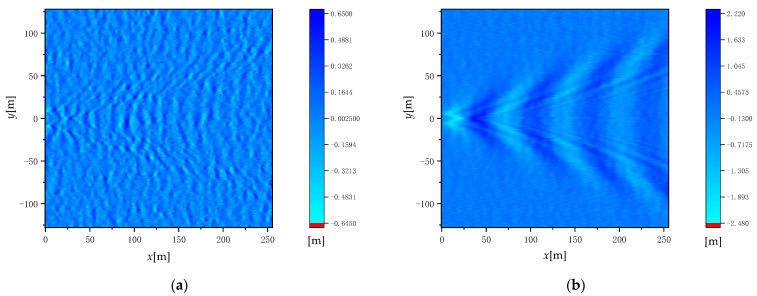
Wave height after linear superposition of time-varying Kelvin wake and time-varying sea surface for (**a**) U_s_ = 5 m/s and (**b**) U_s_ = 10 m/s.

**Figure 6:**
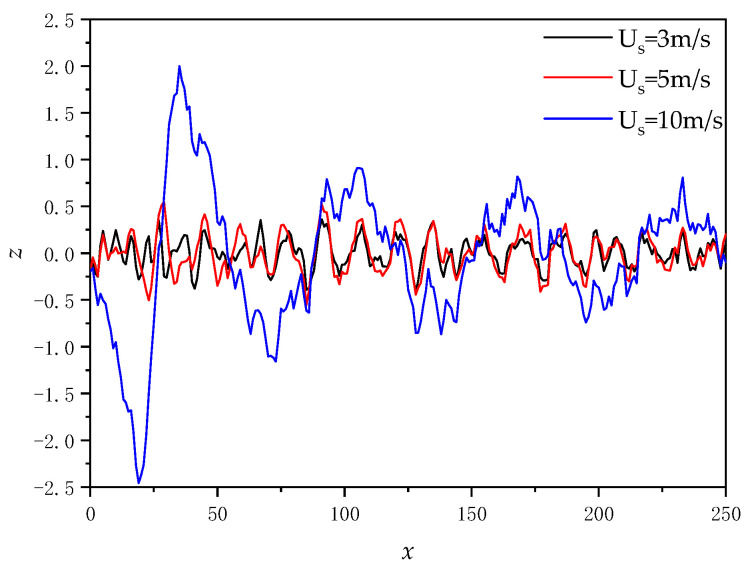
Cross-sectional view of wave heights after linear superposition of the time-varying Kelvin wake and the time-varying sea surface.

Figure 5 illustrates the wave height after linear superposition of time-varying Kelvin wake and time-varying sea surface when the wind speed u = 5 m/s, the wind direction *φ*_w_ = 0°, and the time *t* = 0 (a) for the ship speed U_s_ = 5 m/s and (b) for the ship speed U_s_ = 10 m/s. Figure 6 gives the cross-sectional view of wave heights under the same conditions.

It can be seen from Figure 5 and Figure 6 that with the increase in ship speed, the wave height and wavelength of the wake superimposed with the sea surface increase.

## 3. Facet Scattering Field Model of Sea Surface

**Figure 7:**
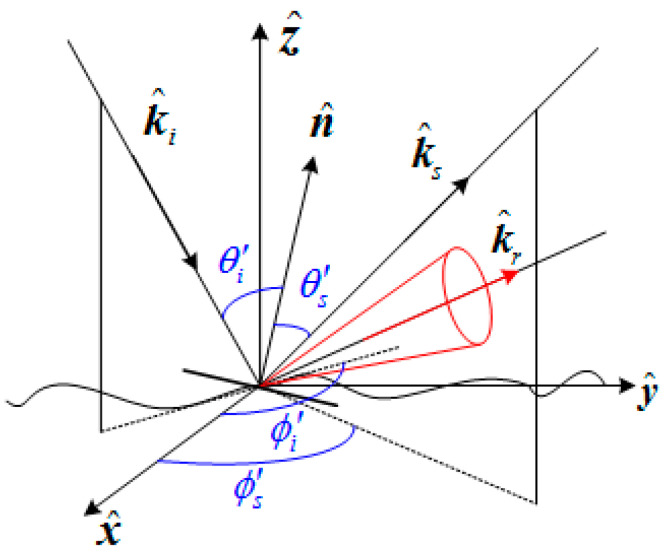
Schematic diagram of specular region.

The scattering area of the two-dimensional sea surface can be divided into the specular region and the diffuse scattering area. The specular region is defined as the inner region of the cone with the mirror direction ${\hat{k}}_{r}$ corresponding to the specific plane element as the central axis and the half-cone angle as 30°, as shown in Figure 7. In other words, if the scattering direction of the incident wave is located on or inside the cone defined above after it is reflected by a local surface element on the sea surface, the scattering of the surface element is considered as specular scattering; otherwise, the scattering of the surface element is considered diffuse scattering. The definition of such a mirrored region can be expressed by the formula:

(13) $$\begin{array}{l} \langle {\hat{k}}_{s},{\hat{k}}_{r}\rangle <20^{{}^{\circ}}\\ {\hat{k}}_{r}={\hat{k}}_{i}-2\hat{n}\left({\hat{k}}_{i}\cdot \hat{n}\right) \end{array}$$

The surface elements that make the main contribution to diffuse scattering are calculated by the facet scattering field model of the sea surface by capillary waves [21,22], and the surface elements that make the main contribution to specular region scattering are calculated by the Kirchhoff approximation (KA) method. Therefore, the scattering field of a single surface element of the sea surface can be expressed as:

(14) $$E_{pq}({\hat{k}}_{i},{\hat{k}}_{s})=\left\{ \begin{array}{l} E_{pq}^{KA}({\hat{k}}_{i},{\hat{k}}_{s}),\;\;specular\;\;region\\ E_{pq}^{facet}({\hat{k}}_{i},{\hat{k}}_{s}),\;non-specular\;\;region \end{array}\right.$$

where

(15) $$\begin{array}{l} E_{pq}^{KA}\left(r\right)=\\ \frac{ik\exp(ikR)}{4\pi R}{\hat{k}}_{s}\times \int \left[(\hat{n}\times E\right)\;-\eta {\hat{k}}_{s}\times (\hat{n}\times H)]\exp[-ik({\hat{k}}_{s}-{\hat{k}}_{i})\cdot r^{\prime }]dS^{\prime } \end{array}$$

(16) $$E_{pq}^{facet}\left({\hat{k}}_{i},{\hat{k}}_{s}\right)=\frac{ik^{2}(\varepsilon -1)\Delta S}{8\pi n_{z}}F_{pq}\frac{e^{ikR}}{R}e^{-iq\cdot r_{0}}\;\left[B(k_{c}^{+})I_{0}(k_{c}^{+})+B(k_{c}^{-})I_{0}(k_{c}^{-})\right]$$

where ${\hat{k}}_{i}$ is the propagation direction vector of the incident electromagnetic wave, ${\hat{k}}_{s}$ is the propagation direction vector of the scattered electromagnetic wave, *k* is the number of electromagnetic waves, $\hat{n}$ is the normal vector, *η* is the intrinsic impedance, *R* is the distance between the center of the plane element and the observation point, **E** and **H** are the total electric field and total magnetic field on the boundary surface, respectively, ∆*S* is the area of the small surface element on the sea surface, ***r***_0_ is the position of the center point of the small plane element, *F_pq_* is the polarization factor, **k**_*c*_ is the wavenumber vector of the capillary component, and *B*(**k**_*c*_) is the amplitude of the capillary wave. The specific expression of *F_pq_*, *B*(**k**_*c*_), and ***I***_0_(**k**_*c*_) can be found in Reference [23].

By summing the scattered fields of all surface elements on the sea surface, the total scattered field on the sea surface can be obtained as follows:

(17) $$E_{pq}^{sea}({\hat{k}}_{i},{\hat{k}}_{s})=\sum_{m=1}^{M}\sum_{n=1}^{N}E_{pq}({\hat{k}}_{i},{\hat{k}}_{s})$$

where *M* and *N* are discrete points of two-dimensional sea surface. The model described in Equation (17) is called the refined facet scattering field model (RFSFM) of sea surface.

**Figure 8:**
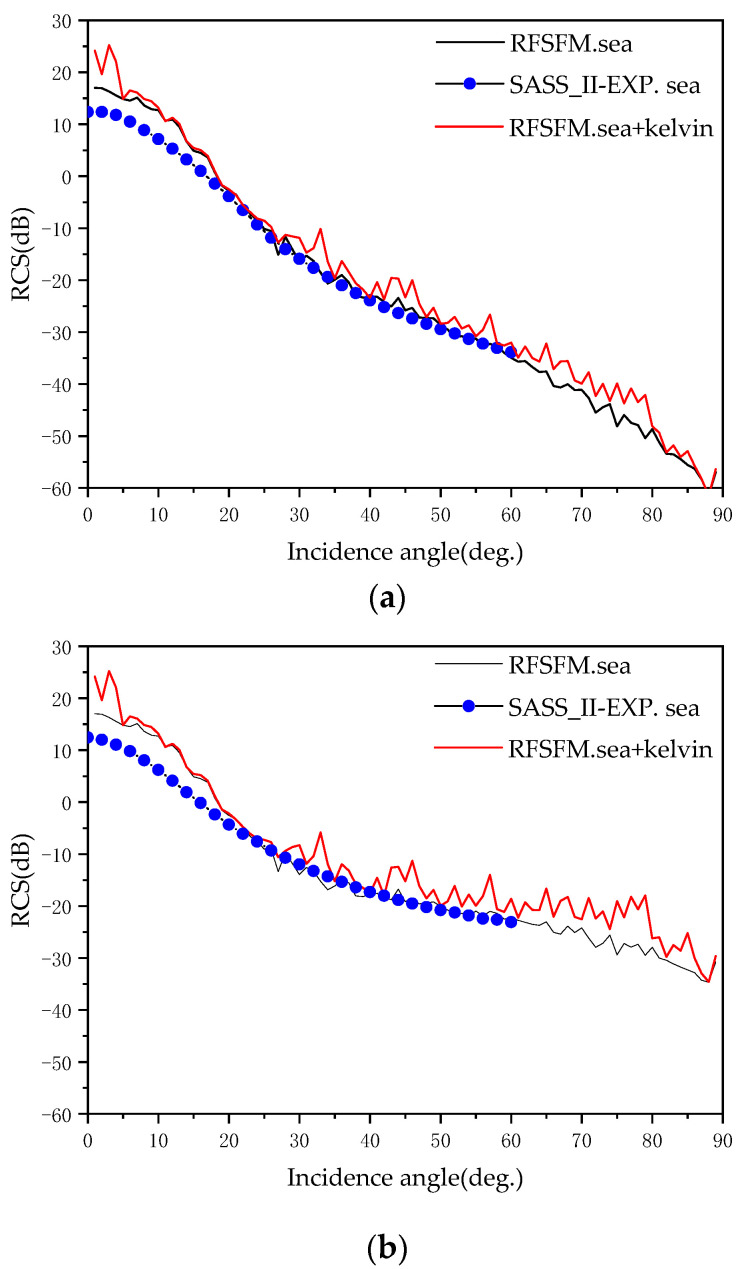
Backscattering coefficients for (**a**) HH polarization and (**b**) VV polarization.

In order to verify the effectiveness of RFSFM, Figure 8 shows the comparison between the backscattering coefficients of pure sea surface calculated by RFSFM and SASS-II data [24]. At the same time, the backscattering coefficients calculated by RFSFM for the sea surface with Kelvin wake are also given (a) for HH polarization and (b) for VV polarization. It can be observed that the backscattering coefficients calculated using RFSFM are in good agreement with the SASS-II data. The parameters required for simulation are shown in Table 2.

It can be seen from Figure 8 that whether HH or VV polarization, on the whole, RCS decreases with the increase in incident angle. At the same time, it can be seen that in part of the interval, the backscattering coefficients of the sea surface with the presence of Kelvin wake wave are larger than that of the pure sea surface. This is because the existence of the wake wave changes the undulation distribution of the sea surface so that the roughness of the sea surface increases, and the scattering effect of the incident wave is enhanced.

**Figure 9:**
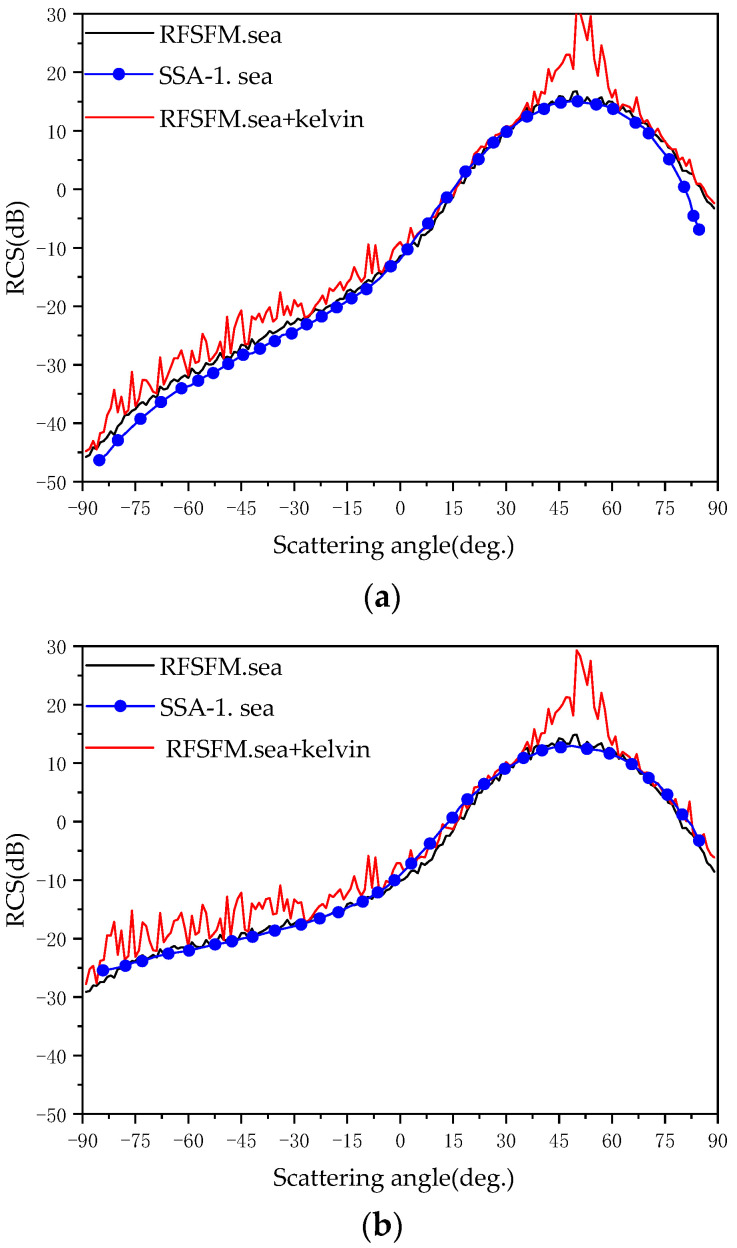
Bistatic scattering coefficients (**a**) for HH polarization and (**b**) for VV polarization.

Figure 9 shows the comparison between the pure sea surface bistatic scattering coefficients calculated by RFFSM and the pure sea surface bistatic scattering coefficients calculated by SSA-1. At the same time, the bistatic scattering coefficients calculated by RFFSM for the sea surface and the Kelvin wake after linear superposition are given (a) for HH polarization and (b) for VV polarization. It can be observed that the results obtained by RFFSM are in good agreement with those obtained by SSA-1. Table 3 provides the parameters used to calculate the bistatic scattering coefficients.

As can be seen from Figure 9, whether HH or VV polarization, the position of the maximum value of RCS appears near the scattering angle equal to the incident angle. Different from the downward trend of backscattering coefficients in the whole interval, bistatic scattering coefficients increase first and then decrease in the interval. After 60°, the RCS curves of the sea surface superimposed with the Kelvin wake almost coincide with the RCS curves of the sea surface without being superimposed with the Kelvin wake under HH polarization. However, there is also a common feature between the two: that is, the RCS of the sea surface with wakes in part of the interval is larger than that of the sea surface alone. 

**Figure 10:**
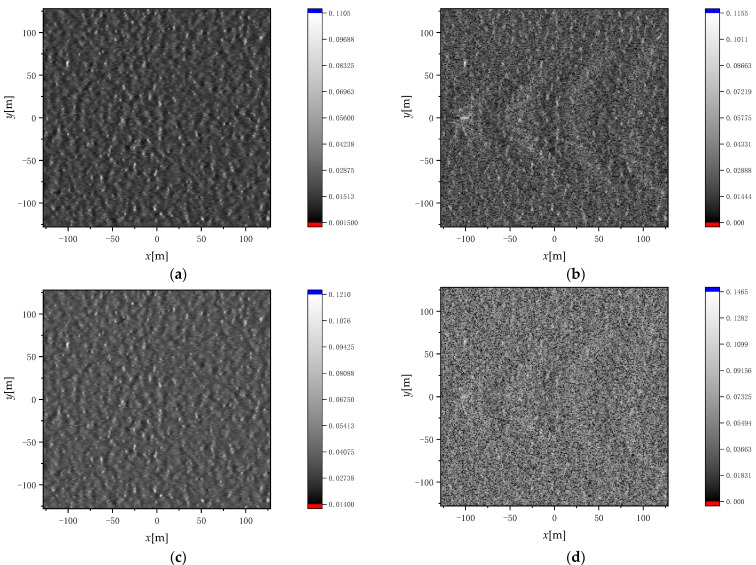
Surface element scattering distribution: (**a**) and (**b**) for HH polarization; (**c**) and (**d**) for VV polarization.

In order to analyze the influence of Kelvin wake on sea surface scattering characteristics from the aspect of detailed features, Figure 10 shows the simulation of surface element scattering distribution of sea surface wake by RFSFM. The radar frequency used in the simulation is 14 GHz, the radar incident angle is fixed at 50°, the wind direction *φ*_w_ = 0°, the wind speed u = 5 m/s, the time t = 0, and the ship speed U_s_ = 10 m/s (a) and (c) for the surface element scattering distribution of pure sea surface, (b) and (d) for the surface element scattering distribution of sea surface after superimposed Kelvin wake, (a) and (b) for HH polarization, and (c) and (d) for VV polarization.

As can be seen from Figure 10, with the surface element scattering distribution of pure sea surface as a reference, the Kelvin wake characteristics can be clearly observed after the Kelvin wake is superimposed. At the same time, it can be observed that HH polarization is better than VV polarization in reflecting the difference between the peak and trough of Kelvin wake.

It is easy to obtain the Doppler spectra of the backscattering echo by the standard spectral estimation method [25], namely

(18) $$S_{0}\left(f\right)=\frac{1}{T}{\left|\int_{0}^{T}E_{pq}^{sea}({\hat{k}}_{i},{\hat{k}}_{s},t)\exp\left(i2\pi ft\right)dt\right|}^{2}$$

where $E_{pq}^{sea}({\hat{k}}_{i},{\hat{k}}_{s},t)$ represents the scattered field of sea surface at time *t*. Equation (18) can be implemented using the fast Fourier transform. In this way, the center frequency *f_c_* can be obtained:

(19) $$f_{c}=\frac{\int fS_{0}\left(f\right)df}{\int S_{0}\left(f\right)df}$$

The classical Bragg frequency shift formula can be obtained from Equation (19), namely:

(20) $$f_{B}=\pm \frac{\sqrt{2gk_{i}\sin\theta_{i}}}{2\pi }$$

where *k_i_* is the wave number of incident electromagnetic waves.

The steps to simulate the Doppler spectrum are as follows:

1. The discrete intervals ∆*x* and ∆*y* of the sea surface were set to simulate the generation of N_t_ samples of the sea surface after the superposition of the ship wake and the sea surface with the time interval τ. If τ is small enough, the sample can be considered stationary at that time interval.
2. RFSFM is used to calculate the scattering field of sea surface samples superimposed by ship wake and sea surface at different time points, to obtain a set of scattering field time series.
3. The Doppler spectra of the obtained scattering field time series are calculated according to the standard spectrum estimation method expressed in Equation (18).
4. Repeat the previous three steps to obtain the calculated results of multiple sets of Doppler spectra and find the average.

If the Doppler spectra of group N_s_ samples are statistically averaged, the average Doppler spectra can be obtained as follows:

(21) $$\bar{S}\left(f\right)=\frac{1}{N_{s}}\sum_{i=1}^{N_{s}}S_{0}^{i}\left(f\right)$$

## 4. Discussion

**Figure 11:**
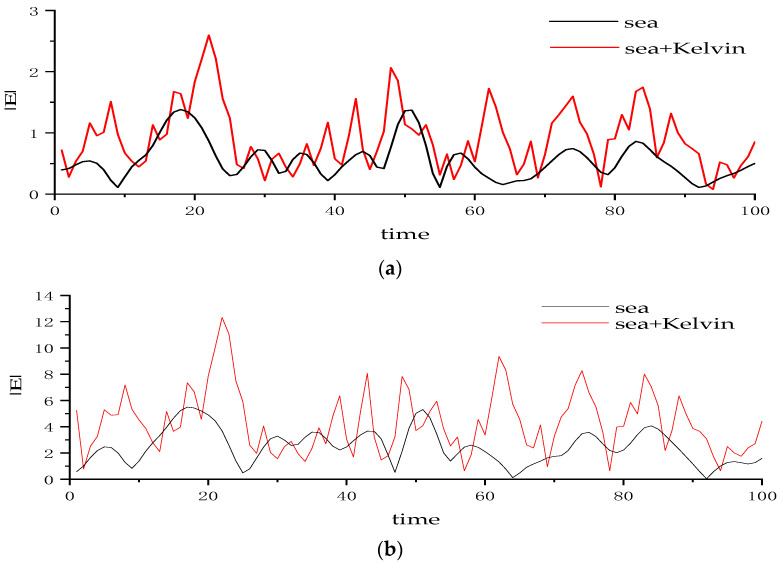
Change of modulus of radar scattering echo with time.

Figure 11 shows the change of the modulus of radar scattering echo with time at ship speed U_s_ = 5 m/s, incident angle θ*_i_* = 60°, wind speed u = 5 m/s, and wind direction *φ*_w_ = 0° (a) for HH polarization and (b) for VV polarization. It can be observed that compared with the echo signal without the wake superimposed on the sea surface, the peak value of the scattered echo signal curve after the wake superimposed on the sea surface increases, and the frequency of the peak value increases.

The simulation of the backscattering Doppler spectrum when the incident electromagnetic wave frequency is 5 GHz will be analyzed below. In the resulting Doppler spectrum, the location of the Bragg frequency is marked by a green vertical line.

**Figure 12:**
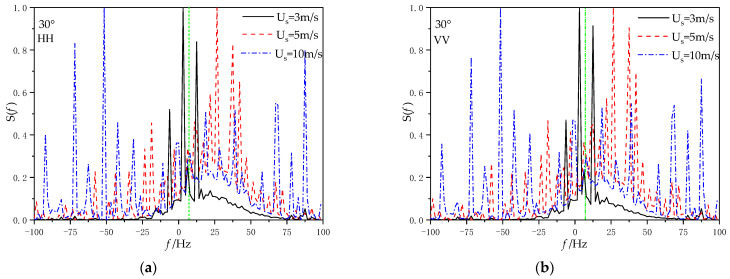

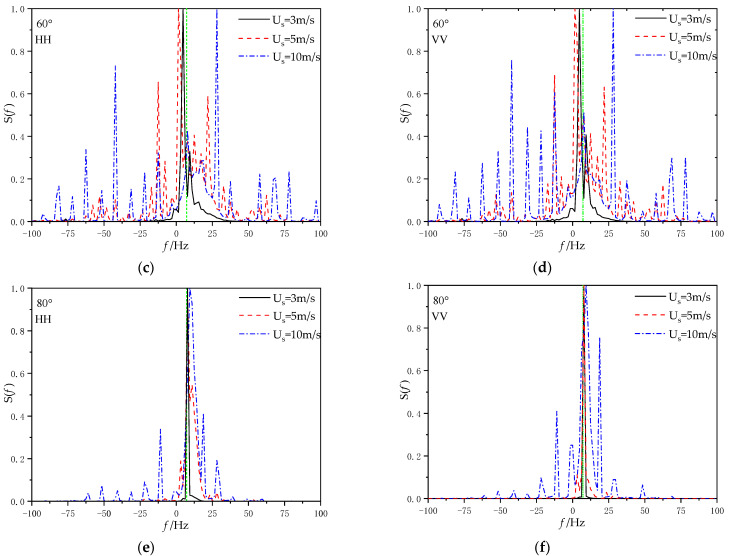
Doppler spectra at different ship speeds: (**a**), (**c**), and (**e**) for the Doppler spectrum at HH polarization; (**b**), (**d**), and (**f**) for the Doppler spectrum at VV polarization.

Figure 12 shows the Doppler spectra of different ship speeds at wind speed u = 5 m/s; wind direction *φ*_w_ = 0°; incident angle θ*_i_* = 30°, 60°, and 80°; ship speed Us = 5 m/s, 8 m/s, and 10 m/s; HH polarization; and VV polarization: (a), (c), and (e) for the Doppler spectrum at HH polarization; (b), (d), and (f) for the Doppler spectrum at VV polarization.

It can be seen from Figure 12 that the main peak of the Doppler spectrum is near Bragg frequency for both HH polarization and VV polarization, and they do not degenerate due to the increase in ship speed, which indicates that the wake motion is modulated by the sea wave. As can be seen from the figure, with the increase in the incidence angle, the peak value of Doppler farther away from Bragg frequency decreases until the spectral peak disappears, while the peak value of Doppler closer to Bragg frequency almost stays the same. At the same time, it can be observed that the Doppler spectrum broadening of sea surface becomes narrower with the increase in incident angle. With the increase in the ship speed, the main peak of the Doppler spectrum widens and creates new peaks on either side of the Bragg frequency. When the ship speed is less than the wind speed, only a few spectral peaks are generated or no new spectral peaks are generated. When the ship speed is close to and greater than the wind speed, multiple new peaks will be generated in the Doppler spectrum regardless of the incident angle. This shows that when the ship speed is less than the wind speed, the wake has little influence on the sea surface roughness; When the ship speed is more than the wind speed, the wake becomes the main factor affecting the sea surface roughness, which is also the reason for the new spectral peak of the Doppler spectrum at large ship speed.

**Figure 13:**
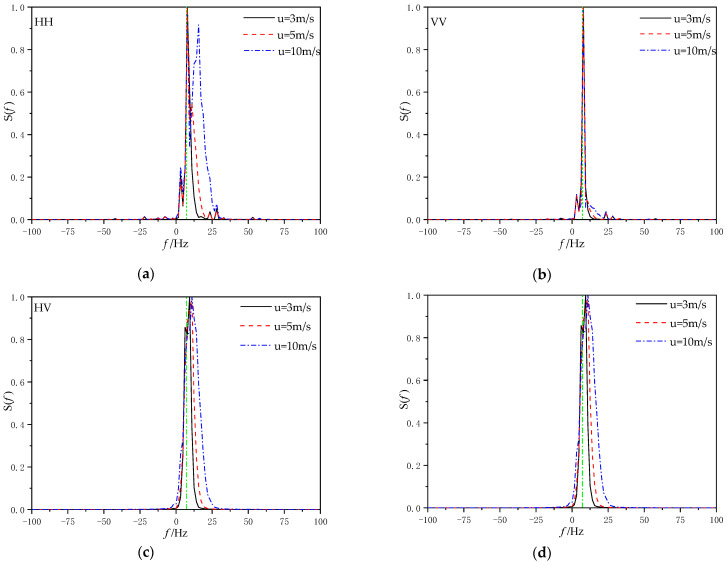
Doppler spectra at different wind speeds for (**a**) Doppler spectrum at HH polarization, (**b**) Doppler spectrum at VV polarization. (**c**) Doppler spectrum at HV polarization, and (**d**) Doppler spectrum at VH polarization.

Figure 13 gives the Doppler spectra at different wind speeds for incident angle θ*_i_* = 80°, wind direction *φ*_w_ *=* 0°, and ship speed U_s_ = 5 m/s (a) for the Doppler spectrum at HH polarization, (b) for the Doppler spectrum at VV polarization, (c) for the Doppler spectrum at HV polarization, and (d) for the Doppler spectrum at VH polarization. 

It can be observed that regardless of the polarization mode, the center broadening of the Doppler spectrum becomes wider with the increase in wind speed. Compared with HH polarization and VV polarization, the Doppler spectrum obtained under cross-polarization only produces a peak near Bragg frequency, but no new peak occurs elsewhere. At the same time, it can be seen that under HH polarization, when the wind speed is close to the sailing speed, a new spectral peak will be generated on the right side of the Bragg frequency, and the peak value of this spectral peak increases with the increase in the wind speed. This indicates that when the wind speed is greater than the ship speed, the sea breeze will dominate the change of sea surface roughness.

**Figure 14:**
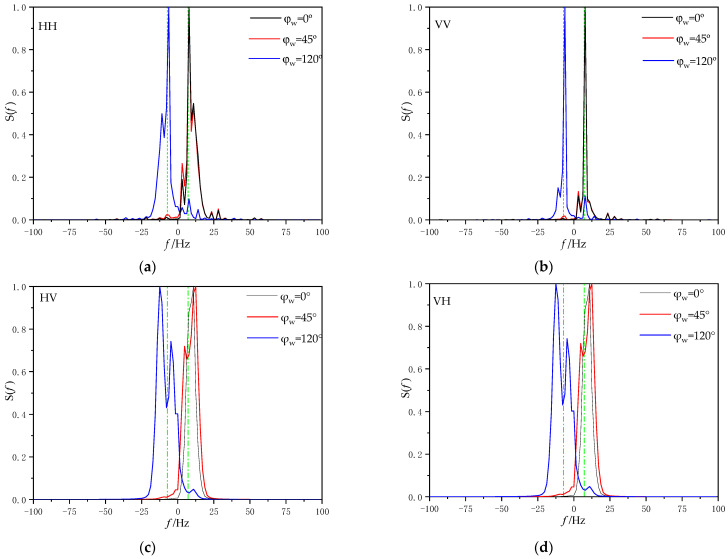
Doppler spectra under different wind directions for (**a**) Doppler spectrum at HH polarization, (**b**) Doppler spectrum at VV polarization, (**c**) Doppler spectrum at HV polarization, and (**d**) Doppler spectrum at VH polarization.

Figure 14 shows the Doppler spectra under different wind directions when the incident angle θ*_i_* = 80°, wind speed u = 5 m/s, ship speed U_s_ = 5 m/s and polarization mode are HH polarization and VV polarization, respectively, (a) for the Doppler spectrum at HH polarization, (b) for the Doppler spectrum at VV polarization, (c) for the Doppler spectrum at HV polarization, and (d) for the Doppler spectrum at VH polarization. 

It can be seen that when the wind direction angle is larger than the incident angle, the Doppler spectrum moves to the negative frequency region. This is because when the wind direction angle is greater than the incidence angle, the sea waves move away from the radar. Compared with VV polarization, the Doppler spectrum broadening under HH polarization is larger than that under VV polarization. Compared with co-polarization, the Doppler spectrum obtained under cross-polarization will generate a new peak on the left side of Bragg frequency with the increase in wind direction angle, and the peak value of this peak will increase with the increase in wind direction angle. 

## 5. Conclusions

The scattering properties of the sea surface superimposed with Kelvin wake are discussed in this paper. RFSFM is used to calculate the static and time-varying scattering fields after a superimposed Kelvin wake. Then, the Doppler of radar scattering echo signal of sea surface wake is simulated under different conditions. The results show that there are obvious differences in Doppler spectra under different sea states. This study provides a reference for extracting motion features of ship wakes in a sea clutter environment. The future investigation will revolve around the EM scattering from a moving ship at a time-varying sea surface with a time-varying ship wake.

## Figures and Tables

**Table 1:** Parameters of the Kelvin wake simulation ship.

Parametric Name	Parametric Value
Ship length	52 m
Ship width	5.9 m
Side wall draft depth	3.5 m

**Table 2:** Parameters required for simulation.

Electromagnetic Wave Frequency	Wind Speed	Wind Direction	Ship Speed	Time
14 GHz	5 m/s	180°	5 m/s	0

**Table 3:** Parameters used to calculate the bistatic scattering coefficients.

Electromagnetic Wave Frequency	Incidence Angle	Wind Speed	Wind Direction	Ship Speed	Time
14 GHz	50°	5 m/s	180°	5 m/s	0

## Data Availability

Not applicable.
